# Pterostilbene enhances sorafenib’s anticancer effects on gastric adenocarcinoma

**DOI:** 10.1111/jcmm.15795

**Published:** 2020-10-13

**Authors:** Tingting Zhao, Chun Wang, Xinying Huo, Ming‐Liang He, Jinfei Chen

**Affiliations:** ^1^ National Clinical Research Center of Kidney Diseases Jinling Hospital Nanjing University School of Medicine Nanjing China; ^2^ Department of Oncology Nanjing First Hospital Nanjing Medical University Nanjing China; ^3^ Department of Biomedical Sciences City University of Hong Kong Kowloon Hong Kong; ^4^ CityU Shenzhen Research Institute Shenzhen China; ^5^ Cancer Center Taikang Xianlin Drum Tower Hospital Nanjing University School of Medicine Nanjing China; ^6^ Jiangsu Key Lab of Cancer Biomarkers, Prevention and Treatment Collaborative Innovation Center for Cancer Personalized Medicine Nanjing Medical University Nanjing China

**Keywords:** combination chemotherapy, gastric adenocarcinoma (GAC), pterostilbene, sorafenib, synergistic effect

## Abstract

Sorafenib has been approved for the treatment of certain cancers in clinic. However, the effects of sorafenib on gastric adenocarcinoma (GAC) were still limited. This study aimed to evaluate both in vitro and in vivo efficacy of sorafenib in combination with pterostilbene (PTE) on the treatment of GAC. Here, the morphological changes and cell viability were recorded in both N87 and MKN45 cells. The cell cycle profile and apoptosis were assessed by flow cytometry. Subcutaneous tumour xenografts were constructed in nude mice, and IHC staining of the dissected tumour tissues was conducted. Our results showed that PTE enhanced sorafenib's inhibitory effects on cell viability. The obvious down‐regulation of cyclin D1, Cdk‐2, Cdk‐4, Cdk‐6 and p62 and the up‐regulation of LC3II, caspase‐9, caspase‐3 and PARP cleavages were observed for the combination treatment with PTE and sorafenib than monotherapy. The combination treatment resulted in a higher level of cell cycle arrest at G1 phase and apoptosis than either drug. Besides, drug combination significantly enhanced the inhibition of tumour growth than sorafenib or PET alone in nude mice. The percentage of Ki‐67‐ and PCNA‐positive cells was distinctly reduced, and the apoptotic cells was obviously increased when compared with single drug therapy. Altogether, PET obviously enhanced sorafenib's antitumour effects against GAC through inhibiting cell proliferation, inducing autophagy and promoting apoptosis. The combination therapy with PET and sorafenib may serve as a novel therapeutic strategy for treating GAC and deserve further clinical trials.

## 1 INTRODUCTION

As the fourth most common and the second most deadly cancer worldwide, gastric adenocarcinoma (GAC) remains one of the major public health problems worldwide.^1^ Nearly two‐thirds of patients recur after curative resection. Currently, chemotherapy followed by surgery is the first‐line treatment for most GAC patients.^2^ Due to drug resistance and severe adverse side effects, combination therapy may be a potential therapeutic approach for GAC patients.^3^ Combination therapy could sensitize GAC cells to the cytotoxic effects induced by monotherapy, reducing the doses of either drug and improving the clinical effects.^4^

Sorafenib, a multi‐kinase inhibitor, has been shown to suppress tumour cell proliferation and induce apoptosis.^5^ It has been approved for the clinical treatment of advanced renal cell carcinoma and unresectable hepatocellular carcinoma.^6^, ^7^ Several other trials against various solid tumours are currently in progress, including gastric cancer, lung cancer, breast cancer and prostate cancer.^8^, ^9^, ^10^, ^11^ Due to the numerous adverse side effects of sorafenib, combination therapies are encouraged in the future investigations to reduce the dosage and improve the clinical therapeutic effects.

Pterostilbene (trans‐3,5‐dimethoxy‐4‐hydroxystilbene, PTE), as a natural dimethylated analogue of resveratrol (RESV) extracted from blueberries, exhibits diverse pharmacologic activities including anticancer, anti‐inflammation, antioxidant, anti‐proliferative and analgesic activities.^12^, ^13^ Under most circumstances, PTE shows more potent antitumour activity than RESV, resulting from the substitution of a hydroxy group with a methoxy group.^14^, ^15^ Therefore, PTE could be more potentially developed for clinical applications.

A recent study showed that sorafenib alone failed to inhibit GAC tumour growth in vivo, while a marked inhibitory effect was induced when co‐administered with non‐toxic diclofenac, an multidrug resistance‐associated protein (MRP) inhibitor.^16^ However, whether pterostilbene would also sensitize the GAC response to sorafenib has never been investigated. In this study, we examined the efficacy of sorafenib and pterostilbene combination on GAC both in vitro and in vivo, and also investigated the underlying mechanism of the enhanced anticancer effects against GAC.

## 2 MATERIALS AND METHODS

### 2.1 Chemicals

**FIGURE 1:**
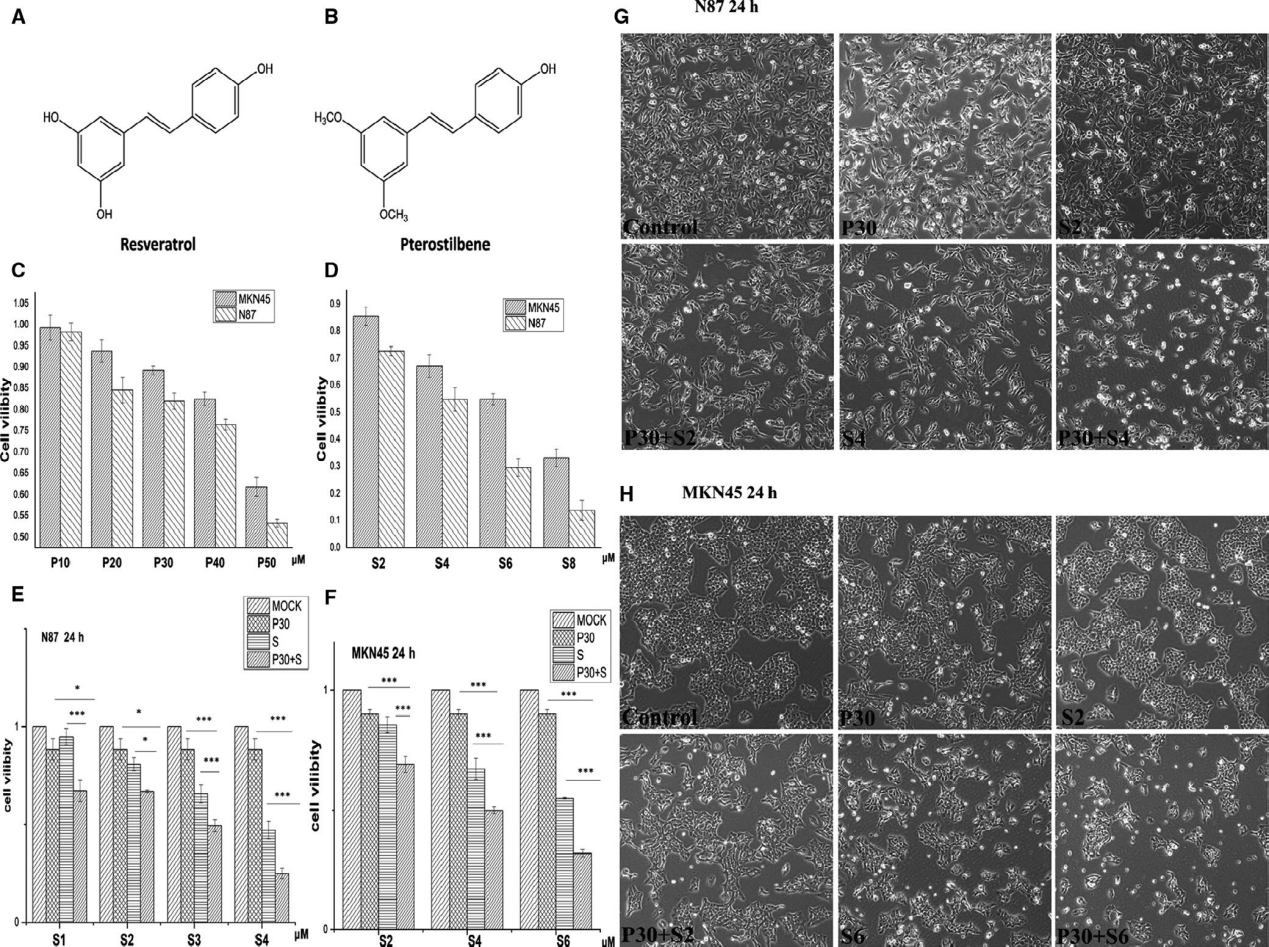
The effects of PTE and sorafenib on cell viability and morphology. (A and B) The chemical structures of resveratrol and pterostilbene. (C‐F) Cell viability was assessed using the MTT assay. N87 and MKN45 cells were treated with PTE (10, 20, 30, 40 and 50 µmol/L) or sorafenib (2, 4, 6, 8 µmol/L) at indicated concentrations for 24 h, respectively (C and D). The combination treatment with PTE (30 µmol/L) and sorafenib (1, 2, 3, 4 µmol/L for N87 and 2, 4, 6 µmol/L for MKN45) for 24 h in N87 (E) and MKN45 cells (F). The result was expressed as a percentage of surviving cell over the control group. Value was shown as mean ± SD of five independent experiments. *P < 0.05, **P < 0.01, ***P < 0.001. (G and H) The morphological changes after 24‐h treatment with sorafenib (2, 4 µmol/L for N87 and 2, 6 µmol/L for MKN45) and/or PTE (30 µmol/L). The cell morphology was observed and recorded under a phase‐contrast microscope in random fields at 200 magnification. P, pterostilbene; S, sorafenib

Sorafenib was purchased from International Laboratory USA (#320790); resveratrol (RESV) was obtained from J&K Scientific Ltd (Woburn, MA, USA), and pterostilbene (PTE) was purchased from Sigma‐Aldrich (St. Louis, MO) with purity over 97% (see structure in Figure 1A,B). All compounds were dissolved in dimethyl sulphoxide (DMSO, Sigma, USA) and further diluted in sterile culture medium immediately prior to the in vitro and in vivo experiments.

### 2.2 Cell lines and cell culture

The study was carried out on two cell lines (N87 and MKN45) derived from human gastric adenocarcinoma. Cells were cultured in Dulbecco's modified Eagle's medium (DMEM) supplemented with 10% foetal bovine serum and 1% penicillin/streptomycin (10 000 units of penicillin/mL and 10 mg/mL streptomycin) in an incubator at 37°C with 5% CO_2_ in air.

### 2.3 Cell viability assay

Cell viability was evaluated using the MTT (3‐(4,5‐dimethylthiazol‐2‐yl)‐5‐(3‐carboxymethoxyphenyl‐2‐(4‐sulfo‐phenyl)‐2H‐tetrazolium) (Sigma‐Aldrich, France) assay. Cells at logarithmic growth period were plated in 96‐well plate at a density of 5000 cells/well in a volume of 100 mL. Then, the cells were treated with target agents at the desired concentrations. Five replicates were conducted for each medication dose. After treated for 24 hours, 10 µL of MTT (10 mg/mL) was added to each well and incubated for another 4 hours. After abandoning the supernatants, 100 µL of DMSO (Sigma, USA) was added to each well to dissolve the crystals. Subsequently, the optical density (OD) of each well was measured using a microplate reader at a wavelength of 550 nm (BMG Labtech, Ortenberg, Germany). The 50% inhibitory concentration (IC_50_) values were calculated using the GraphPad Prism^®^ 5 (version 5.01, GraphPad Software, Inc, USA). The percentage of cell viability was calculated based on the following formula: cell viability (%) = [1 − (average absorbance of experimental group/average absorbance of blank control group)] × 100%.

### 2.4 Western blot assay

Western blot assay was used to detect protein expression levels. First, cells were lysed in radioimmunoprecipitation assay (RIPA) buffer (25 mmol/L Tris∙HCl pH7.6, 150 mmol/L NaCl, 1% NP‐40, 0.25% sodium deoxycholate, 0.1% SDS) with protease inhibitor cocktail (Roche Diagnostics, Mannheim, Germany). Then, the protein lysates were denatured at 95°C for 5 minutes after mixing with 5x SDS‐loading buffer. Subsequently, the cell extracts (30 µg protein) were separated on a sodium dodecyl sulphate‐polyacrylamide electrophoretic gel (SDS‐PAGE) and then transferred to nitrocellulose membranes. After blocked with 3% BSA for 2 hours, the membranes were incubated overnight at 4°C with the following primary antibodies at dilutions of 1:1000: PARP (#9532), Bcl‐2 (#2876), Bax (#14796), Caspase‐9 (#9502), Caspase‐3 (#9664), CyclinD1 (#2978), CDK2 (#2546), CDK4 (#12790), CDK6 (#13331), CyclinD1 (#2978) purchased from Cell Signaling Technology, and PCNA (#ab29) obtained from Abcam. Next, the membrane was incubated with the corresponding horseradish peroxidase–labelled secondary antibody (Santa Cruz Biotechnology) for 2 hours at room temperature. Lastly, the signal was visualized by an enhanced chemiluminescence (ECL) kit (Immobilon Western HRP, MILLIPORE, USA) according to the manufacturer's instructions.

### 2.5 Cell cycle assay

Cells (2 × 10^5^ cells/well) were seeded in 6‐well plate to adhere overnight and then synchronized by starvation for 3 days. After the treatment with agents at the desired concentrations for 16 hours, cells were harvested and fixed in ice‐cold 75% ethanol at 4°C overnight. After washed with PBS, the cells were stained with propidium iodide (PI, 40 µg/mL) and RNAase (50 µg/mL) in dark for 30 minutes at room temperature. Subsequently, the samples were detected by flow cytometry (BD Biosciences, San Jose, CA, USA) and the cell cycle profiles were analysed using the ModFit LT Program (Verify Software House, Topsham, ME, USA).

### 2.6 Annexin V/PI apoptotic assay

Annexin V‐FITC apoptosis detection kit (BD, San Jose, USA) was used to measure the apoptotic cells. Cells were harvested, washed twice with ice‐cold PBS and re‐suspended in Annexin V binding buffer at a concentration of 1 × 10^6^ cells/mL. Subsequently, 5 μL Annexin V and 5 μL PI were added and then incubated in dark for 15 minutes at room temperature. Finally, 400 μL binding buffer was added to each tube before analysing the percentage of apoptotic cell by flow cytometer. Five replicates were analysed for each dosage, and the data were processed by CellQuest software (BD Biosciences).

### 2.7 Xenografts

N87 cells (3 × 10^5^ cells) were suspended in 0.1 mL of PBS and then inoculated subcutaneously into the right flank of five‐week‐old male BALB/c nude mice. When the average tumour size reached approximately 100 mm^3^, the animals were randomly distributed into the following four groups (n = 4/group) and administered with: (a) control (100 mL PBS, daily, intraperitoneal injection), (b) PTE (250 mg/kg, dissolved in 100 mL PBS, every other day, intraperitoneal injection), (c) sorafenib (30 mg/kg, dissolved in 20 mL corn oil, daily oral) and (d) PTE plus sorafenib (administered as described for single‐agent treatment). The dosage adopted in our experiment has been shown to have prominent antitumour efficacy in the previous tumour xenograft models. The tumour volume and the mice bodyweight were measured every 3 days. Tumour volumes were calculated using the formula (l × (w)^2^)/2, in which l was the longest length in dimension and w was the width of the tumour. Follow‐up was terminated at day 35 after inoculation. The mice were killed by carbon dioxide euthanasia. After recording the tumour weight, the tumour tissues were fixed in 4% paraformaldehyde for immunohistochemistry.

### 2.8 Immunohistochemistry

Firstly, the dissected tumour tissues were formalin‐fixed, paraffin‐embedded and sectioned. Next, the sections were deparaffinized in xylene, rehydrated through the descending grades of alcohol and then washed with PBS. Antigen retrieval was performed with citrate buffer (pH 6.0) for 20 minutes in microwave, and then the activity of endogenous peroxidase was quenched with 3% H_2_O_2_ for 30 minutes. After blocking with BSA, the slides were incubated with PCNA and Ki67 (Santa Cruz Biotechnology, sc‐15402) overnight at 4°C at 1:50 dilution, and then incubated with the appropriate secondary antibody for 1 hour at room temperature. Besides, the TUNEL assay was conducted to detect apoptotic cells according to the protocol as described by the manufacturer (Beyotime Biotechnology). The IHC staining was visualized by 3,3′‐diaminobenzidine (DAB), followed by counterstaining with haematoxylin (Sigma). Five random optical fields from each section were recorded at ×200 magnifications under Nikon Eclipse E400 microscope. And the number of positive staining cells was counted for each field.

### 2.9 Statistical analyses

All analyses were performed with the software SPSS ver. 20.0 (SPSS Inc, Chicago, IL, USA). Data were presented as mean ± standard deviation (SD) in at least five independent experiments. *t* Test and two‐way ANOVA was used for all the statistical analyses. A *P* value less than 0.05 was considered statistically significant.

## 3 RESULTS

### 3.1 Cell viability and morphological changes

The differences in the chemical structures between RESV and PTE were the substitution of a hydroxy group with a methoxy group (Figure 1A,B). First, N87 and MKN45 cells were treated with indicated concentration of PTE or RESV for 24 hours and then subjected to MTT assay for cell viability. As shown in Table 1, PTE showed a stronger cell growth inhibitory effect than RESV. The IC_50_ value was 52.71 ± 1.23 μmol/L vs 116.68 ± 2.45 μmol/L for N87 cells and 65.63 ± 1.52 μmol/L vs 132.56 ± 2.38 μmol/L for MKN45 cells, respectively. Therefore, PTE was selected for combination studies. Figure 1C,D shows that PTE and sorafenib significantly inhibited cell viability in a dose‐dependent manner. In N87 cells, the IC_50_ of PET and sorafenib was 52.71 ± 1.23 μmol/L and 3.85 ± 0.23 μmol/L, respectively. In MKN45 cell, the IC_50_ of PET and sorafenib was 65.63 ± 1.52 μmol/L and 6.27 ± 0.34 μmol/L, respectively (Table 1).

**TABLE 1:** The cytotoxicity induced by PTE, sorafenib or RESV after 24‐h treatment

Agent	PET		Sorafenib		RESV	
Cell	N87	MKN45	N87	MKN45	N87	MKN45
IC50 (μM)	52.71 ± 1.23	65.63 ± 1.52	3.85 ± 0.23	6.27 ± 0.34	116.68 ± 2.45	132.56 ± 2.38

Note

The values represented the mean ± SD of five independent experiments.

Abbreviations: PET, pterostilbene; sorafenib; RESV, resveratrol.

Next, to examine whether combination treatment displays better anticancer effects, PTE was used at a lower concentration of 30 μmol/L (near half of IC_50_), which induced limited cell growth inhibition (80%‐90% of viability) over 24 hours. In addition, N87 cells were treated with sorafenib at concentrations of 1, 2, 3 and 4 μmol/L, and MKN45 cells were treated with sorafenib at 2, 4 and 6 μmol/L (Figure 1E,F). The combination of PTE and sorafenib significantly increased the inhibition of cell viability than monotherapy, and the synergistic interaction was enhanced in a sorafenib dose‐dependent manner (Figure 1E,F). The morphological changes induced by the mono‐ or combined treatments in N87 and MKN45 cells are shown in Figure 1G,H. Cells treated with PTE alone were characterized with few autophagic vacuole accumulations in cytoplasm, and no obvious morphological changes were observed among those treated with sorafenib along beside cell density reduction. Importantly, the combination of PET and sorafenib showed more prominent autophagic vacuole formation, cell volume loss, chromatic condensation and nuclear fragmentation. In addition, much more death cells floated on the culture surface in the combined treatment group (Figure 1G,H).

### 3.2 The enhanced effects on cell cycle arrest in G1 phase by PTE

**FIGURE 2:**
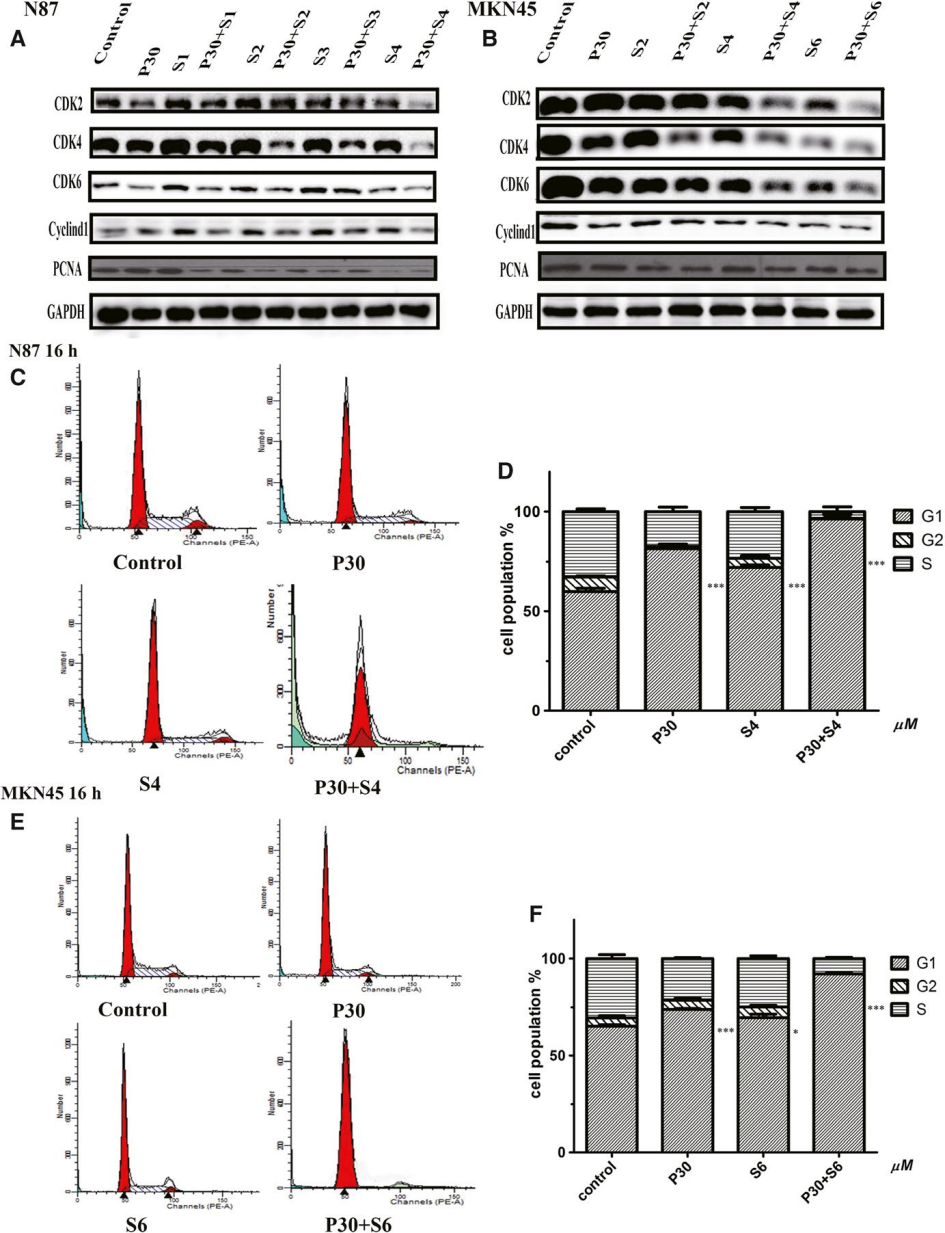
The effects of PTE and sorafenib on cell cycle arrest in G1 phase. (A and B) The down‐regulation of cell cycle–related proteins after 16‐h treatment with the indicated concentrations of sorafenib and/or PTE. The protein levels of cyclin D1, CDK2, CDK4, CDK6 and PCNA were detected by Western blot assays in N87 (A) and MKN45 cells (B); and GAPDH was used as the loading control. (C‐F) Cell cycle profiles were determined by flow cytometry assays after 16‐h treatment with sorafenib (4 µmol/L for N87 and 6 µmol/L for MKN45) and/or PTE (30 µmol/L). The representative cell cycle distribution images were chosen for N87 (C) and MKN45 (E) cells. Data from five independent experiments were statistically analysed and graphically depicted for N87 (D) and MKN45 (F) cells, respectively. *P < 0.05, **P < 0.01, ***P < 0.001 for G1 phase as compared with control

As a marker of proliferation, PCNA expression was significantly decreased upon combined treatment when compared with PTE or sorafenib alone (Figure 2A,B). In order to reveal whether cell proliferation inhibited by PTE and sorafenib was caused by cell cycle arrest, we monitored the expression levels of the cell cycle regulatory proteins. Our results showed that the protein levels of cyclin D1, Cdk‐2, Cdk‐4 and Cdk‐6 in both N87 and MKN45 cells were obviously down‐regulated in the combination treatment groups than that in the monotherapy groups (Figure 2A,B). Similar to the cell viability, the effects on the expression of cell cycle regulatory proteins were also enhanced in a sorafenib dose‐dependent manner (Figure 2A,B). Besides, results from flow cytometry assays showed that treatment with either sorafenib (4 μmol/L for N87 and 6 μmol/L for MKN45) or PET (30 μmol/L) alone induced cells accumulated in G1 phase with a concomitant decrease of cells in S phase after 16 hours treatment. As expected, the G1 phase accumulation was significantly higher in the combination treatment group than that in the monotherapy ones, confirming the enhanced growth inhibitory effects induced by cell cycle arrest (*P* < 0.01 Figure 2C‐F).

### 3.3 The enhanced effects on the promotion of apoptotic and autophagy by PTE

**FIGURE 3:**
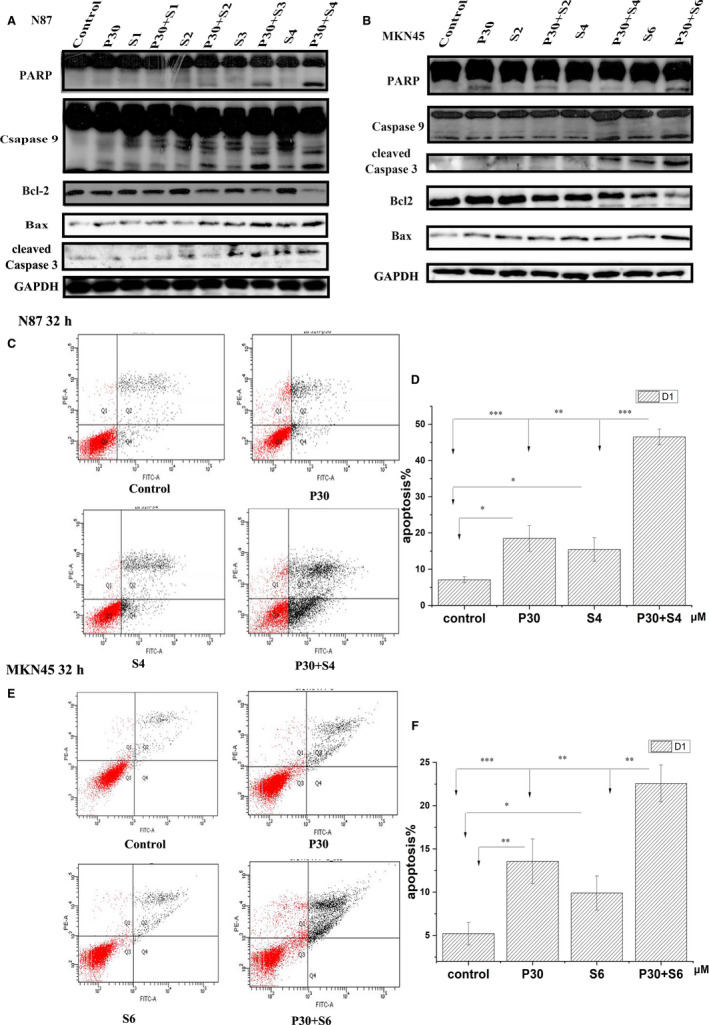
The effects of PTE and Sorafenib on apoptosis. (A and B) The protein level of apoptotic marker proteins after 32‐h treatment with the indicated concentration of sorafenib and/or PTE. The apoptotic markers PARP, caspase‐3, caspase‐9, Bax and Bcl‐2 were detected in N87 (A) and MKN45 cells (B) by Western blot assay; and GAPDH was used as the loading control. (C‐F) The percentage of apoptotic cells was evaluated by flow cytometry assay after 32‐h treatment with sorafenib (4 µmol/L for N87 and 6 µmol/L for MKN45) and/or PTE (30 µmol/L). The representative Annexin V/PI double‐staining images were chosen for N87 (C) and MKN45 (E) cells. The percentage of Annexin V–positive cell calculated from five independent experiments was statistically analysed and graphically depicted for N87 (D) and MKN45 cells (F). *P < 0.05, **P < 0.01, ***P < 0.001

**FIGURE 4:**
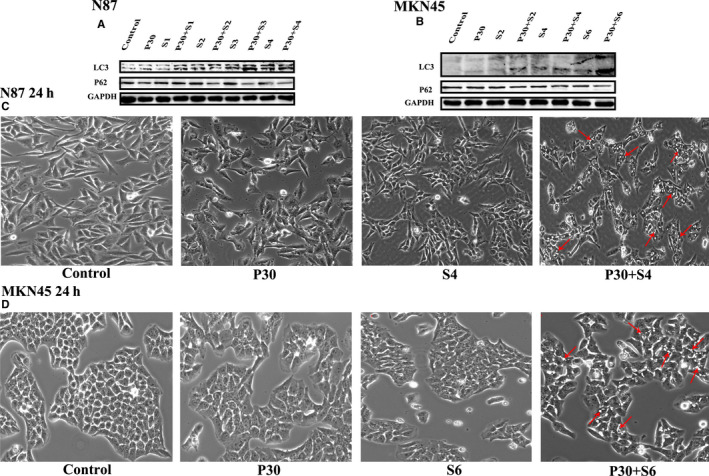
The effects of PTE and sorafenib on autophagy. (A and B) The expression level of key regulators of autophagy after 24‐h treatment with the indicated concentrations of sorafenib and/or PTE. LC3‐II and p62 were detected in N87 (A) and MKN45 cells (B) by Western blot assays; and GAPDH was used as the loading control. (C and D) The autophagic vacuole formation after 24‐h treatment with sorafenib (4 µmol/L for N87 and 6 µmol/L for MKN45) and/or PTE (30 µmol/L). The representative images of autophagic vacuoles were observed and recorded under a phase‐contrast microscope in random fields at 200 magnification for N87 (C) and MKN45 (D) cells. The autophagic vacuoles were marked with red arrows

As shown in Figure 3A,B, PET and sorafenib did not affect the expression level of and induce cleavage of caspase 3, caspase 9 and PARP at the test concentrations. Compared with each mono‐treatment, the combination of PTE and sorafenib dramatically promoted the cleavage of apoptotic‐related proteins including caspase‐9, caspase‐3 and PARP. Meanwhile, the expression level of the anti‐apoptotic protein Bcl‐2 significantly decreased and the pro‐apoptotic protein Bax obviously increased in response to the combined treatment. And the cytotoxicity was enhanced in a sorafenib dose‐dependent manner (Figure 3A,B). In addition, the effects of PTE and sorafenib on apoptosis were further investigated by a standard Annexin V–based apoptosis assay. In this assay, the dead cells are stained by propidium iodide, while the early apoptotic cells are stained by FITC‐conjugated Annexin V. They can be well separated and counted by a flow cytometer. In N87 cells, a significantly higher percentage of apoptotic cells were observed in the group of combined treatment with 4 μmol/L sorafenib and 30 μmol/L PTE (46.5 ± 2.17%) for 32 hours than that in the monotherapy groups (PTE: 18.5 ± 3.54%, sorafenib: 15.4 ± 3.25%, respectively) (*P* < 0.01; Figure 3C,D). Similar results were also obtained from MKN45 cells. The combination treatment led to 26.2 ± 1.2% apoptotic cells as compared with 7.8 ± 0.78% in sorafenib (6 μmol/L) and 9.8 ± 1.27% in PTE (30 μmol/L) treatment groups (*P* < 0.01; Figure 3E,F). Next, we compared the different effects on autophagy between the combined treatment group and the monotherapy ones. Our results showed that the expression of p62 was significantly decreased and the level of LC3II was obviously increased as compared with mono‐treatment (Figure 4A,B). In addition, the morphological studies demonstrated that few autophagic vacuole accumulations in cytoplasm was observed with PTE or sorafenib treatment alone, but much more prominent autophagic vacuoles formed upon combination treatment (Figure 4C,D). We speculated that autophagy may also be involved in the anticancer enhancement. In summary, our results demonstrated PTE enhanced sorafenib's effects on GAC by the inhibition of cell cycle and the induction of apoptosis and autophagy.

### 3.4 The enhanced antitumour effects by PTE in xenograft models

**FIGURE 5:**
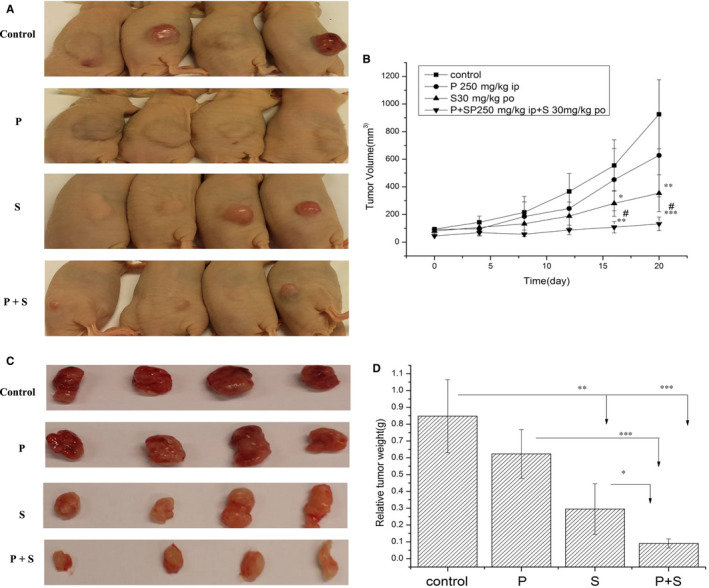
The antitumour effects of the combination therapy with PTE and sorafenib in vivo. (A) Photographs showing the morphological characteristics of the N87 xenografts in 4 groups. When the transplanted tumour volume reached approximately 100 mm^3^ (3 × 10^6^ N87 cells, subcutaneous injection), the mice were randomly distributed into four groups (n = 4/group) and administered with: 1) vehicle (100 mL/PBS, ip, daily), 2) PTE (250 mg/kg, ip, every other day), 3) sorafenib (30 mg/kg, po, daily) and 4) PTE (250 mg/kg, ip, every other day) plus sorafenib (30 mg/kg, po, daily) for 3 weeks. (B) The tumour growth curve of N87 xenografts after treatment was drawn with the tumour volume measured every 3 days. *P < 0.05, **P < 0.01, ***P < 0.001 for the comparison with vehicle group; ^#^ *P* < 0.05 for the comparison with sorafenib group. (C and D) The image of the dissected tumour tissues was shown (C) and the statistical analysis of tumour weight measured at the endpoint of experiment (D). Data from four independent experiments were statistically analysed and present as the mean ± SD

To evaluate the antitumour effects and the therapeutic safety of the PET and sorafenib combination in vivo, we constructed subcutaneous tumour xenografts using N87 cells in athymic nude mice. All treatment schemes were well tolerated and no apparent adverse effects (eg fatigue, mortality, significant weight loss, skin toxicity, hepatotoxicity, nephrotoxicity and cardiac toxicity) were observed (Figure 5A; Table 2; Table S1). As shown in Figure 5B,C, the PET or sorafenib monotherapy moderately inhibited tumour growth as compared with control (*P* < 0.05). More importantly, the tumour volume of the group treated with PET and sorafenib displayed significant inhibitory effect as compared to either agent alone or vehicle control groups (*P* < 0.05; Figure 5B). Consistent with the tumour volume, the tumour weight of the mice co‐treatment with PET and sorafenib was also obviously lower than those of the single drug‐treated or untreated groups (*P* < 0.05; Figure 5D).

**TABLE 2:** The antitumour effects of sorafenib with or without PET in N87 xenograft model

Treatment	No. of mice	Tumour volume (mm^3^)	Tumour weight (g)	Bodyweight (g)
Control (100 mL, ip, daily)	4	925.25 ± 249.82	0.85 ± 0.22	20.61 ± 0.61
PTE (250 mg/kg, ip, every other day)	4	627.82 ± 300.10	0.62 ± 0.14	20.32 ± 0.36
Sorafenib (30 mg/kg, po, daily)	4	353.84 ± 133.21	0.30 ± 0.15	20.57 ± 0.81
PTE (250 mg/kg, ip, every other day) + sorafenib (30 mg/kg, po, daily)	4	132.27 ± 49.24	0.09 ± 0.03	20.12 ± 0.49

Note

The results were expressed as the mean ± SD.

Abbreviations: ip, intraperitoneal injection; po, orally.

**FIGURE 6:**
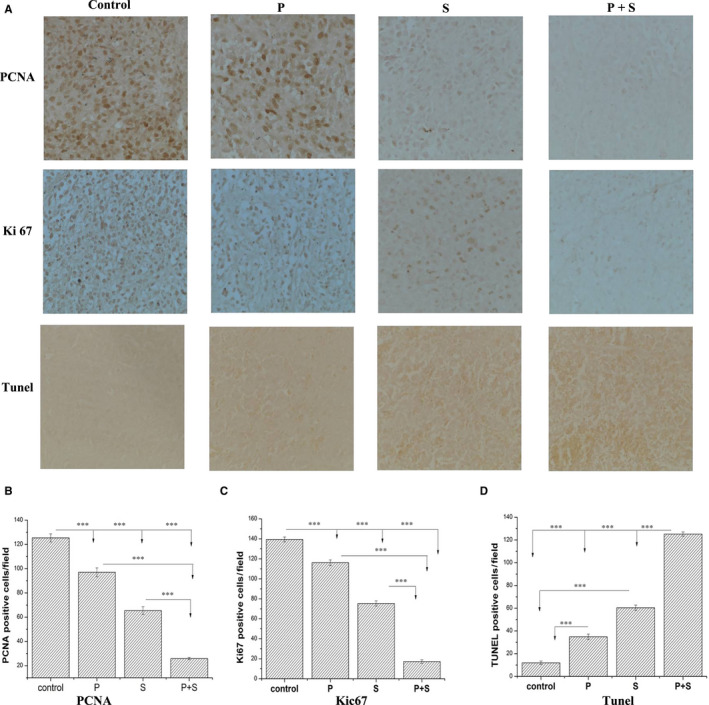
The IHC verification of the enhanced antitumour effects in dissected tumour tissues by combination therapy. (A) The representative IHC images (200×) of the proliferative cells stained with anti‐Ki67 and anti‐PCNA; and the apoptotic cells stained with TUNEL in vehicle, PET, sorafenib and PET plus sorafenib groups were shown. The experiments were repeated three times with similar results. (B‐D) The effects on proliferation inhibition and apoptosis promotion were verified by IHC staining in vivo. Treatment with PET plus sorafenib resulted in obviously decreased PCNA (B) and Ki67 (C) positive cells and increased TUNEL‐positive cells (D) than monotherapy. The number of positive staining cells was counted from five random (200×) fields. *P < 0.05, **P < 0.01, ***P < 0.001

The IHC scoring of the dissected tumour tissues revealed that the percentage of Ki‐67‐ and PCNA‐positive cells was distinctly reduced in the co‐treatment group with PET and sorafenib than those in single‐agent treatment groups (Figure 6A‐C). TUNEL staining assay also showed that the number of apoptotic cells in the group received combined treatment was obviously higher than those accepted monotherapy (Figure 6A,D). Above all, our in vivo results demonstrated that the combination therapy with PET and sorafenib resulted in much better antitumour effects through the decrease of proliferation and the elevation of apoptosis, as compared with monotherapy and vehicle control groups.

## 4 DISCUSSION

Sorafenib exhibited obvious anticancer effects on GAC cell lines with numerous adverse side effects.^17^, ^18^ The combination with another antitumour agent may display a better clinical prospect with reduced sorafenib dose.^19^ PTE is a potential anticancer agent for clinical applications.^14^ For the first time, our present investigation demonstrated that PTE distinctly enhanced the anticancer activity of sorafenib both in vitro and in vivo by inducing G0/G1 cell cycle arrest, apoptosis and autophagy.

We demonstrated that sorafenib and PET inhibited N87 and MKN45 cell proliferation in a dose‐dependent manner. PET potentiated sorafenib‐mediated cytotoxicity as evidenced by more prominent morphological changes including autophagic vacuole formation, cell volume loss, chromatic condensation, nuclear fragmentation, cell density reduction, cell death and so on (Figure 1). In vivo, the enhanced antitumour effects were also observed by reduced tumour volumes and weights (Figure 5). Besides, the Ki‐67 and PCNA IHC staining of the dissected tumour tissues were also distinctly reduced in the co‐treatment group (Figure 6).

Cell proliferation is regulated by cell cycle, and the loss of cell cycle checkpoint control is a hallmark in cancer progression.^20^, ^21^ Our present study demonstrated that PTE potently enhanced the sensitivity of cancer cells to sorafenib by inducting cell cycle arrest at G1 phase (Figure 2C‐F). Besides, more obvious down‐regulation of cell cycle activators (cyclin D1, Cdk‐2, Cdk‐4 and Cdk‐6) was observed in the combination treatment group as compared with the mono‐treatment groups (Figure 2A,B). During cell division, cyclin D1 forms a complex with CDK4/CDK6 to promote DNA replication in cell cycle progression.^22^ Oppositely, the decreased expression of cyclin D1/Cdk4/Cdk6 inactivates pRb, causing cell cycle arrest in G1 phase and the inhibition of cell proliferation.^23^, ^24^

There are two major types of programmed cell death (PCD), that is apoptosis and autophagic death.^25^, ^26^ Our results showed that PET effectively sensitized GAC cells to sorafenib induced apoptosis via the intrinsic mitochondrial pathway. PTE combined with sorafenib dramatically promoted the cleavages of apoptotic‐related proteins than monotherapy, including caspase‐9, caspase‐3 and PARP (Figure 3A,B). Cytochrome c released from mitochondria to cytosol first forms apoptosome with procaspase‐9 that further induces caspase‐3 activation and PARP cleavage.^27^, ^28^, ^29^ Caspase‐3, an ultimate executioner of the caspase family, is responsible for the nuclear changes during apoptosis including chromatin condensation.^30^ PARP, a highly conserved nuclear enzyme, plays significant roles in DNA repair, recombination, proliferation and genomic stability.^31^, ^32^ Besides, the permeabilization of cytochrome c is also regulated by anti‐apoptotic proteins (Bcl‐2, Bcl‐xl, Mcl‐1) and pro‐apoptotic proteins (Bax, Bad, Bak).^33^, ^34^ Bcl‐2 inhibits the oligomerization of Bax, leading to inhibition of cytochrome c release.^35^ In our study, we demonstrated that the combination treatment strongly increased the Bax/Bcl‐2 ratio than mono‐treatment (Figure 3A,B). So, the apoptosis induced by mitochondrial intrinsic pathway may be another explain for the enhanced anticancer effects by PTE, which were also been proven in vivo by the TUNEL staining in the tumour tissues (Figure 6A,D).

Autophagy is characterized by the accumulation of autophagic vacuoles or autophagosomes, followed by fusion with lysosomes to form autophagolysosomes and subsequent degradation of their intracellular organelles.^36^, ^37^ We observed much more autophagic vacuoles in the cancer cells upon combination treatment (Figure 4C,D). The key event of autophagy is the conversion of LC3 from LC3‐I (18 kD cytosolic free form) to LC3‐II (16 kD autophagosomal membrane‐bound form).^38^ Meanwhile, P62/sequestosome1⁄SQSTM1, as a cargo protein binding to LC3‐II and ubiquitinated proteins, is sequestered inside the autophagosome and degraded by autophagy.^39^, ^40^, ^41^ Our results showed that the combination therapy with PET and sorafenib more strongly activated autophagy than monotherapy, evidenced by the up‐regulation of LC3‐II and down‐regulation of p62, as compared with single‐agent treatment alone (Figure 4A,B). In addition, the down‐regulation of Bcl‐2 could also apparently promote autophagy.^42^

In conclusion, our results demonstrated that PET potently enhanced sorafenib's antitumour effects. PTE potently enhanced the sensitivity of GC cells to sorafenib by the inhibition of cell proliferation, promotion of apoptosis and induction of autophagy, allowing for the lower doses of sorafenib and reducing adverse side effects. Due to the limited dose tested in this study, more dosage combinations should be done before setting up clinic trials. We believe that the combination therapy with PET and sorafenib may serve as a novel therapeutic strategy for the treating GAC patients and deserve further clinical trials.

## CONFLICT OF INTEREST

The authors declare that they have no competing interests.

## AUTHOR CONTRIBUTIONS

**tingting zhao:** Data curation (lead); Investigation (lead); Methodology (lead); Writing‐original draft (lead). **chun wang:** Conceptualization (equal). **xinying huo:** Data curation (equal). **jinfei chen:** Resources (lead); Supervision (lead). **mingliang he:** Supervision (equal); Writing‐review & editing (equal).

## ETHICS APPROVAL AND CONSENT TO PARTICIPATE

Animal studies were performed in accordance with the criteria outlined in the ‘Guide for the Care and Use of Laboratory Animals’ prepared by the National Academy of Sciences and published by the National Institutes of Health (USA). Five‐week‐old male BALB/c nude mice were maintained under specific pathogen‐free conditions and manipulated according protocols approved by the Committee on the Ethics of Animal Experiments of the Nanjing Medical University.

## Supporting information

Table S1

## Data Availability

The data sets used and/or analysed during the current study are available from the corresponding author on reasonable request.
