# Supplementary material for: Pterostilbene enhances sorafenib’s anticancer effects on gastric adenocarcinoma
Source: J Cell Mol Med. 2020 Oct 13;24(21):12525–36. doi: 10.1111/jcmm.15795 (PMC7686996; doi:10.1111/jcmm.15795)
Supplement: Supplementary file 1 — Table S1 [file JCMM-24-12525-s001.docx]

**Supplementary Table S1: Toxicity profile of sorafenib with or without PET in N87 xenograft model**

| Treatment | hepatotoxicity | | nephrotoxicity | | cardiac toxicity | |
| --- | --- | --- | --- | --- | --- | --- |
|  | **ALT**  **(U/L)** | **AST**  **(U/L)** | **BUN**  **(mmol/L)** | **CREA**  **(mmol/L)** | **TnT**  **(ng/ml)** | **TnI**  **(ng/ml)** |
| Control (100ml, ip, daily) | 36.71±13.28 | 87.35±20.16 | 7.91±1.39 | 75.39±14.78 | 0 | 0 |
| PTE (250mg/kg, ip, every other day) | 36.75±11.31 | 88.29±23.53 | 7.93±1.17 | 75.62±12.31 | 0 | 0 |
| Sora (30mg/kg, po, daily) | 36.89±17.25 | 89.32±19.31 | 8.05±1.72 | 76.31±16.52 | 0 | 0 |
| PTE (250mg/kg, ip, every other day) + Sora (30mg/kg, po, daily) | 37.27±15.19 | 89.61±20.70 | 8.16±1.93 | 76.87±11.69 | 0 | 0 |

PTE, pterostilbene; Sora, sorafenib; ip, intraperitoneal injection; po, orally; the results were expressed as the mean ± SD.
